# Non-long terminal repeat (non-LTR) retrotransposons: mechanisms, recent developments, and unanswered questions

**DOI:** 10.1186/1759-8753-1-15

**Published:** 2010-05-12

**Authors:** Jeffrey S Han

**Affiliations:** 1 Department of Embryology, Carnegie Institution of Washington, Baltimore, MD, USA

## Abstract

Non-long terminal repeat (non-LTR) retrotransposons are present in most eukaryotic genomes. In some species, such as humans, these elements are the most abundant genome sequence and continue to replicate to this day, creating a source of endogenous mutations and potential genotoxic stress. This review will provide a general outline of the replicative cycle of non-LTR retrotransposons. Recent findings regarding the host regulation of non-LTR retrotransposons will be summarized. Finally, future directions of interest will be discussed.

## Introduction

The biological drive to replicate makes it almost inevitable that selfish genetic elements will populate genomes [1]. Indeed, genome sequencing has revealed that single copy genes are often vastly outnumbered by repetitive transposable elements [2-6]. The abundance and distribution of any particular transposon depends on how aggressive the transposon is (how quickly it can multiply in copy number), where the transposon inserts new copies, and how the host responds. Since these factors can vary greatly, the transposon content of each species is unique and virtually impossible to predict *a priori*. In humans, this number is at least 45% [2].

Transposons can be divided into two broad classes: DNA transposons and retrotransposons. DNA transposons replicate via a cut and paste mechanism [7], whereas retrotransposons replicate using an RNA intermediate. Retrotransposons can be further subdivided into long terminal repeat (LTR) and non-LTR retrotransposons. LTR retrotransposons are retroviral-like in structure and mechanism [8]. Non-LTR retrotransposons (also called LINEs, polyA retrotransposons, or target-primed (TP) retrotransposons), as implied by their name, do not contain LTRs and instead take on the likeness of an integrated mRNA. They are ancient genetic elements that have persisted in eukaryotic genomes for hundreds of millions of years [9], and are perhaps best known for their enormous success multiplying in the human genome. Although non-LTR retrotransposons can be parasitized by non-autonomous elements (for example, short interspersed transposable elements (SINEs)), this review will focus on autonomous elements, which encode the protein machinery necessary for their self-mobilization.

### Non-LTR retrotransposons: general structure

**Figure 1:**
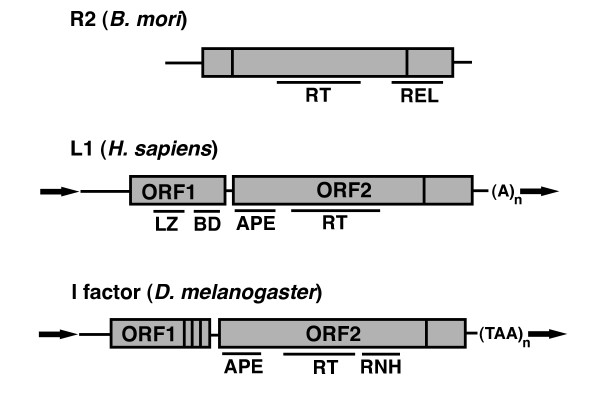
**Structure of non-long terminal repeat (non-LTR) retrotransposons**. The general structures of three model non-LTR elements are shown. Top: the *Bombyx mori *R2 element consists of a single open reading frame (ORF) containing reverse transcriptase (RT) activity and a restriction enzyme-like (REL) domain. Vertical lines indicate cysteine-histidine rich regions believed to encode nucleic acid binding domains. Horizontal lines are untranslated regions. Middle: the human L1 element contains two ORFs. ORF1 contains a leucine zipper (LZ) domain involved in protein-protein interactions and a C-terminal nucleic acid binding domain (BD). ORF2 has a N-terminal apurinic/apyrimidinic endonuclease (APE), a central RT domain, and a C-terminal cysteine-histidine rich domain. The element ends with a polyA tail. Typical insertions are flanked by target site duplications (bold arrows). Bottom: the *Drosophila *I factor element. ORF1 contains three C-terminal cysteine-histidine rich motifs resembling those of retroviral *gag*. ORF2 contains an APE endonuclease, central RT/RNaseH (RNH) domain, and C-terminal cysteine-histidine domain. In all structures contiguous gray boxes represent a single ORF.

Full length, autonomous non-LTR retrotransposons typically contain one or two open reading frames (ORFs). The general structure of three model examples, the *Bombyx mori *R2 element, the human L1 element, and the *Drosophila melanogaster I *factor, are depicted in Figure 1. Central to retrotransposon mobilization is reverse transcriptase (RT) activity, and thus all autonomous non-LTR retrotransposons contain an RT domain. Also present in virtually all non-LTR retrotransposons is an endonuclease (endo) domain [10-12], although encoded endo activity is not an absolute requirement for non-LTR retrotransposition (see Mechanisms below). A second ORF (ORF1) appears to have been an early evolutionary addition [12]. ORF1 contains RNA binding activity [13-15] and nucleic acid chaperone activity [16], and may play a similar role to the *gag *proteins of retroviruses.

The 5' and 3' untranslated regions (UTRs) of non-LTR retrotransposons are quite variable. Although most characterized 5' UTRs of functional non-LTR retrotransposons contain internal promoter activity [17-25], promoter replacement is frequent [26,27] and therefore promoter sequence is not necessarily conserved among elements from different species. Evidence suggests that these promoters are transcribed by RNA polymerase II. There are also elements with no apparent endogenous promoter. For example, the R2 elements, which are found in insect ribosomal DNA loci, do not appear to encode their own promoter, and are thought to be cotranscribed with their host rRNA repeat [28]. The 3' UTR of non-LTR elements usually contains a specific sequence/structure that is recognized by the reverse transcriptase ORF [29-32]. A notable exception is the mammalian L1 element, for which the 3' UTR is dispensable for retrotransposition [33]. The 3' boundary of non-LTR retrotransposons can consist of polydeoxyadenosine (polyA) sequence, short sequence repeats [30,34], or neither. Because of the wide variability in the UTRs of non-LTR retrotransposons, we hesitate to make sweeping generalizations on specific details of non-LTR transcription initiation and termination. Although Figure 1 shows full-length elements, non-LTR retrotransposons in genomes are often 5' truncated due to incomplete reverse transcription [35]. Only a subset of elements are full length and active; for example, out of 500,000 L1s in the human genome, only approximately 7,000 are full length, and of those only 80 to 100 are estimated to be active for retrotranposition [36].

### Non-LTR retrotransposon replication mechanisms

#### Outline of life cycle

**Figure 2:**
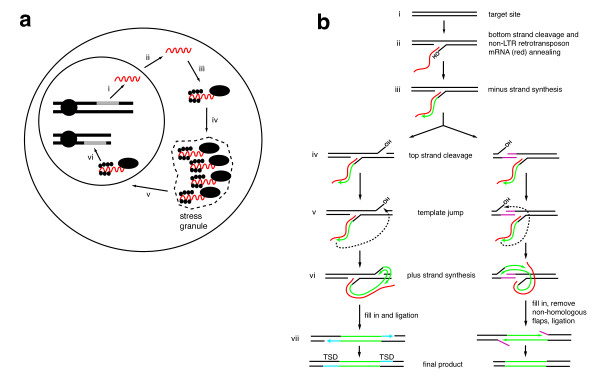
**Replication of non-long terminal repeat (non-LTR) retrotransposons**. **(a) **Replicative cycle of non-LTR retrotransposition. (i) Transcription of a full-length, active element. (ii) mRNA export from the nucleus. (iii) Translation of retrotransposon proteins and (iv) passage through cytoplasmic granule. (v) Ribonucleoprotein particle (RNP) import into the nucleus. (vi) Integration via target-primed reverse transcription (TPRT). **(b) **A model for TPRT. (i) Original unmodified target site. This sequence at this site will vary depending on the specificity of the retrotransposon endonuclease. (ii) Cleavage of one strand of the target site (bottom strand in the figure) by endonuclease. (iii) Minus strand synthesis using retrotransposon mRNA as a template. (iv) During or after minus strand synthesis, top strand cleavage occurs by the retrotransposon endonuclease or a cellular endonuclease. A downstream cleavage (left pathway) leads to a target site duplication (TSD). An upstream cleavage (right pathway) leads to a target site deletion. (v) Template jump from mRNA to top strand of target site. (vi) Plus strand synthesis using the cleaved top strand as primer. (vii) Left pathway: complete of synthesis and fill in of gaps (blue) leads to a target site duplication. Right pathway: completion of synthesis and degradation of non-homologous flaps by unknown nucleases leads to a target site deletion (purple sequences). There are alternative products that are not shown in this model for simplicity.

A general outline of non-LTR retrotransposon replication is shown in Figure 2a. Following transcription and nuclear export, the ORF(s) are translated and assemble to form a ribonucleoprotein particle (RNP) [37-39]. For pol II transcribed elements, the first ORF is most likely translated by the traditional cap recognition and scanning model. This mode of translation (as opposed to an internal ribosomal entry site (IRES)) is supported by experiments in mammals demonstrating that the 5' UTR, 3' UTR, and interORF sequence of L1 are dispensable for retrotransposition ([33]; JSH and Jef Boeke, Johns Hopkins University, unpublished results), the coding region of ORF1 can be extensively mutated without compromising retrotransposition [40], and that translation initiated from the human L1 5' UTR is cap dependent [41]. Elements transcribed by pol I, such as the R1/R2 elements, may be translated via an IRES [28], although this awaits experimental confirmation. In bicistronic transcripts, translation of the second ORF2 relies upon unconventional translation initiation. Studies with the silkworm SART1 element and human L1 element suggest similar translation mechanisms, where ORF2 translation is dependent on ORF1 translation [42,43]. The ribosome that translated ORF1 may reinitiate translation at ORF2, or may recruit another ribosome to serve this purpose.

Using ORF1 as a marker, non-LTR retrotransposon RNPs in most cases are localized predominantly in the cytoplasm [37,39,44-51]. An interesting exception has been reported in rat, where endogenous rat L1 ORF1 protein is concentrated in the nucleus in a chronic myeloid leukemia (CML) cell line [52]. The reason for this anomalous expression pattern is unclear, although one plausible explanation is the production of a mutated version of ORF1 in this particular cell line that escapes cytoplasmic retention [50,52]. Furthermore, a cloned, retrotranspositionally competent rat L1 produced predominantly cytoplasmic RNPs when localized in other cell lines [52]. Other notable examples of nuclear non-LTR retrotransposon RNPs are the telomere targeted elements, which are likely beneficial to the cell [53,54].

Closer examination of non-LTR retrotransposon RNPs in mammals has revealed localization to stress granules, cytoplasmic bodies closely associated with P bodies [50]. Stress granules have been described as a repository for non-translating mRNAs [55]. It is not clear whether stress granule-associated non-LTR RNPs are destined for destruction, or whether transit through the stress granule is an important step in the maturation of a retrotransposon RNP. However, it is noteworthy that loss of P body components in yeast lead to a decrease in Ty1 and Ty3 (LTR retrotransposons) activity, suggesting an important role of P bodies and/or stress granules in the life cycle of retrotransposons [56-60]. The dynamics of non-LTR retrotransposon RNPs have been studied most dramatically with the *Drosophila I *factor [39,47]. *I *factor RNPs appear in nurse cells then are translocated to the oocyte cytoplasm along with other nurse cell components. In the oocyte, the RNPs migrate from the posterior to anterior cortex, following the microtubule organizing center and the nucleus [39,47]. *I *factor RNP migration is reminiscent of *bicoid *mRNA/Staufen protein RNP transport [47,61,62]. The microtubule dependence of this process suggests that non-LTR RNPs may also track along the cytoskeleton. There are also hints that L1 ORF1 protein interacts with intermediate filaments, further bolstering this hypothesis [52]. At some point, a non-LTR retrotransposon RNP must have access to a chromosome, and therefore is presumably transported into the nucleus [63,64], where reverse transcription occurs.

#### Target-primed reverse transcription

Once at a suitable chromosomal target site, a non-LTR retrotransposon can begin to copy its genetic information at this new locus. The mechanism for this process has been termed target-primed reverse transcription (TPRT) and the initial stages have been demonstrated most convincingly with *in vitro *studies of the *Bombyx mori *R2 element. Purified R2 protein, when mixed with R2 RNA and a suitable DNA target site, is able to nick the DNA on one strand (the bottom strand in Figure 2b). The resulting free 3' DNA end serves as a primer to reverse transcribe the R2 RNA (minus strand synthesis). A similar system using human L1 ORF2 has also provided evidence for this reaction, although products could not be monitored directly due to their low yield and non-uniformity (L1 ORF2, unlike the R2 ORF, does not nick a rigidly defined target DNA). The target site of non-LTR retrotransposons is dictated by the encoded endonuclease domain. Ancient non-LTR clades, such as CRE, R2, and R4, encode restriction enzyme-like endonucleases [12] that recognize specific sequences. This endo domain was replaced by an abasic endonuclease early in non-LTR evolution [12], leading in most cases to relaxed target site specificity. The importance of the endonuclease domain lies in producing a primer for reverse transcription. If a suitable primer can be created via another mechanism, non-LTR retrotransposition can proceed in an endo-independent manner [65]. Perhaps the most dramatic example of endo-independent non-LTR retrotransposition occurs naturally in *Drosophila*, where the codependent non-LTR retrotransposons TART, HeT-A and TAHRE retrotranspose at chromosome ends to form the telomeres [54,66-68]. A similar reaction has been recapitulated on mammalian chromosome ends with an engineered endo mutant human L1 [69], suggesting the possibility of a 'pre-endo' era of non-LTR retrotransposons that extended and/or repaired free DNA ends.

The remaining steps of non-LTR integration remain somewhat murky but are hypothesized to occur as follows (see Figure 2b). After or during minus strand synthesis, a second strand nick of the target site occurs (on the top strand of Figure 2b). Depending on the specific non-LTR retrotransposon involved, the second strand nick can occur downstream, upstream, or in line with the bottom strand nick to generate target site duplications (TSDs), target site deletions, or blunt insertions. The non-LTR endo domain is presumed although not proven to also make this second nick, and evidence suggests that at least one non-LTR RT/endo ORF works as a dimer, with each subunit catalyzing one of the nicks [70,71]. How the position of the second strand nick is selected is not entirely clear and probably differs between different elements. In some cases, the second strand nick is likely predefined by the retrotransposon endo domain. For mammalian elements, there is some evidence that microhomology to the newly synthesized bottom strand dictates second strand cleavage [72,73]. In most cases, however, we simply do not know how the second strand cleavage occurs.

Second strand cleavage creates a primer for plus strand synthesis. The newly synthesized minus strand jumps templates from the retrotransposon RNA to the target site DNA top strand. This strand exchange is possibly facilitated by the addition of non-templated nucleotides [74] and/or the assistance of the ORF1 nucleic acid chaperone activity [16]. Plus strand synthesis proceeds from the second nick. Some non-LTR clades have acquired an RNase H domain, which likely removes mRNA template as minus strand synthesis occurs [12,75]. Others have no evidence of an RNase H domain, and the mRNA may simply be displaced by second strand synthesis [76]. In the case of a downstream second strand nick, gaps are filled in to produce TSDs (Figure 2b, left pathway). In the case of an upstream second strand nick (Figure 2b, right pathway), non-homologous flaps are removed by unknown factors to generate target site deletions. After DNA synthesis is complete, the remaining nicks are ligated to complete the insertion.

### Host-encoded factors facilitating non-LTR retrotransposons

It is generally believed that host-encoded factors are required for a complete integration (TPRT) reaction [77]. There are multiple reasons for this line of thought. No non-LTR retrotransposon has ever been shown (biochemically or bioinformatically) to encode proteins with all the expected abilities required to complete such a reaction. For example, the nicks generated by TPRT need to be sealed after DNA synthesis, and encoded ligase activity has not been found within non-LTR elements. In the case of target site deletions, unidentified nucleases eliminate unpaired DNA (Figure 2b, vii, right pathway). In addition, the complete retrotransposition event has never been recapitulated *in vitro *with purified substrate and non-LTR proteins. This of course could simply be due to the complex multistep nature of non-LTR integration and our inability to find the appropriate test tube conditions. However, sequence analysis of genomic integration events suggests the involvement of host DNA repair machinery in some instances of non-LTR integration [72,78]. Finally, experimental data suggests a dependence of non-LTR retrotransposons on host DNA repair pathways. Transfection of a human L1 plasmid into mammalian cells leads to the generation of endodependent DNA double strand breaks (DSBs), and successful L1 retrotransposition in this tissue culture system depends on the ATM kinase involved in homologous recombination and non-homologous end joining (NHEJ) [79]. More recently, cells deficient in the NHEJ factors Ku70, Artemis, and LigIV showed partial inhibition of retrotransposition product formation [80]. Similar studies of the *Lactococcus lactis *L1.LtrB group II intron have revealed multiple *Escherichia coli *DNA repair factors required for intron retrohoming [81]. These bacterial introns share a common ancestry with non-LTR retrotransposons and thus are highly relevant to understanding non-LTR mechanisms [9]. Overall, these data implicate host factors in the resolution of TPRT; however, the limitations of the assays used allow alternative explanations (see Lingering questions below). Non-LTR retrotransposons are not unique in this regard, as other transposon classes also require host factors for resolution of transposition intermediates and/or repair of the donor site.

### Host regulatory mechanisms limiting non-LTR retrotransposons

From the perspective of a transposon, it makes sense to be most active in the germ line or sexual phase of the host organism's life cycle; after all, these are the cells that will give rise to the next generation. In contrast, transposition events in somatic cells will be evolutionarily non-productive, since those new transposition events are lost when the host organism dies. As would be predicted, non-LTR retrotransposons are expressed and retrotranspose in germ cells [45-47,82]. While transposon 'success' depends on transposition in the germ line, the genomic insult of excess transposition can have negative effects on the host. Therefore, host cells generally repress transposon activity. This implies that germ cells are a major evolutionary battleground between transposons and the host, where loss of transposon repression can have dramatic consequences. For example, hybrid dysgenesis is a long known phenomenon in *Drosophila *where the progeny of transposon-naïve females and active transposon-containing males are subject to a range of syndromes including mutation, chromosomal rearrangements, and sterility [83,84]. This syndrome is due to the host's inability to control transposon activity. More recently, the devastating effect of unleashing excess transposition in the mammalian germ line has become apparent, where upregulation of L1 and IAP elements are postulated to lead to sterility [85-88].

#### Small RNA pathways

The discovery of RNA interference (RNAi) [89] led to the hypothesis that small RNAs are involved in silencing transposons via DNA methylation and degradation of transposon RNA. Forward genetic screens revealed that RNAi mutants can indeed lead to general transposon derepression [90,91]. We now know that cells express various classes of small RNAs: microRNAs (miRNAs), endogenous small interfering RNAs (endo-siRNAs), and Piwi interacting RNAs (piRNAs) are among the most studied. A significant fraction of endo-siRNAs and piRNAs correspond to transposon sequence, implying a sequence-based recognition and repression mechanism [92-107]. These RNAs interact with Argonaute protein family members in order to carry out their regulatory functions [108]. piRNAs are germ cell specific, while endo-siRNAs are ubiquitous.

A model for piRNA-mediated silencing of transposons in the germ line has emerged from elegant experiments in *Drosophila *and mice. In this model, two classes of piRNAs associate with different Argonaute family members. A class of piRNAs corresponding to the sense orientation of transposon transcripts associates with Piwi and Ago3 (MILI in mice) [85,94], and a class of piRNAs antisense with respect to transposon mRNA associates with Aubergine (MILI2 in mice) [94,109]. These classes of piRNAs are amplified by a 'ping-pong' mechanism whereby the sense strand piRNA directs cleavage and formation of an antisense strand, and *vice versa *[94]. The origin of the antisense piRNAs, in many cases, can be traced to long piRNA precursors transcribed from a small number of discrete piRNA clusters in the genome [94], some of which are already known to be 'master regulators' of transposon control [110-116].

Mutation of endo-siRNA and piRNA Argonaute partners leads to transposon upregulation [85-87,117-119]. Transposon upregulation is associated with defects in germ cell development, emphasizing the importance of transposon silencing [86-88]. In germ cells, ping-pong amplification is presumed to not only cleave transposon mRNA leading to post-transcriptional repression, but also serves to generate sequence-specific substrates to guide DNA methylation. It is well known that transposons are associated with DNA methylation, and this methylation plays an important role in long-term transposon silencing [120,121]. Mutations in MILI and MIWI2 lead not only to piRNA defects but also failure of proper transposon DNA methylation, providing a genetic link between piRNA production and the eventual methylation of transposon DNA [87]. It is assumed that the piRNA effector complexes somehow recruit the *de novo *DNA methyltransferases DMNT3A, DMNT3B, and their associated protein DMNT3L to methylate transposon loci. How this is accomplished is not yet clear, and the molecular mechanisms underlying these processes are currently an area of active investigation [122-124]. It is likely that this process will resemble well studied pathways of heterochromatin formation in various organisms (reviewed in [125,126]). We should also mention that these mechanisms are not specific to non-LTR retrotransposons but apply to all transposons.

#### Intracellular defense by the APOBEC protein family

The antiviral activity of the APOBEC3G cytidine deaminase was first recognized for its ability to inhibit the HIV retrovirus [127,128]. APOBEC3G cytosine deamination of the newly synthesized HIV cDNA strand results in G to A hypermutation in the viral coding sequence. Since retroelements synthesize a similar nascent cDNA strand, this finding led to the hypothesis that APOBEC family members similarly inhibit retrotransposons. Indeed, all APOBEC3 family members have been shown to inhibit retrotransposon activity [129-135] using a cell line-based assay, although there are inconsistencies in the literature as to whether APOBEC3G inhibits L1 elements. These inconsistencies may be due to varying levels of endogenous APOBEC3 proteins expressed in laboratory cell lines [135]. In addition, the *activation-induced deaminase *(AID) gene, which may share common ancestry with the APOBEC3 proteins, also inhibits L1 in the same cell line-based assay [136].

Surprisingly, hypermutation of the integrated retrotransposition product does not occur upon APOBEC3 member coexpression with non-LTR retrotransposons, and cytidine deaminase activity was shown to be dispensable for the antiretrotransposon effect in most but not all cases (a separation of function of antiviral activity from cytidine deaminase activity has also been shown for HIV [137]). Thus, there are likely at least two distinct mechanisms for non-LTR retrotransposon inhibition by APOBEC family members: deaminase dependent and deaminase independent. The lack of coding strand G to A hypermutation in the deaminase-dependent mechanism could be due to the quick degradation of nascent cDNA minus strands containing uracil, preventing the resolution of deaminase-modified retrotransposition intermediates. The mechanism of deaminase-independent APOBEC3 inhibition of non-LTR retrotransposons is currently unclear, although localization of the specific proteins may give clues to possible function. APOBEC3A, B, C, and H, inhibitors of human L1, localize at least partially to the nucleus [131,135], suggesting a possible function in blocking L1 integration. APOBEC3DE, F, and G appear predominantly in the cytoplasm. They may serve a role in cytoplasmic sequestration of RNPs, although a fraction of these APOBECs may also shuttle to and perform a function in the nucleus [131]. Recently APOBEC3G has been shown to localize to P granules and stress granules [138]. Given the presence of L1 RNPs in stress granules [50], APOBEC3G may sequester L1 in these granules. Coexpression of ORF1 protein with Alu (a non-autonomous non-LTR parasite of L1) shifts a fraction of Alu transcripts from the nucleus to ORF1p-containing stress granules [51]. Interestingly, APOBEC3G also inhibits Alu elements [139,140], and the colocalization of APOBEC3G, L1, and Alu suggest a common (but poorly understood) mechanism.

It should be noted that APOBEC3 proteins have recently been implicated in the clearance of foreign DNA introduced into cultured cells, irrespective of whether the foreign DNA encodes a transposable element [141]. This restriction of foreign DNA also has both deaminase-dependent and deaminase-independent pathways, and perhaps casts some doubt on the specificity of APOBEC retroelement control. However, these data are somewhat at odds with controls performed in prior studies [132,142]. Ultimately, definitive proof for biologically relevant regulation of non-LTR retrotransposons by APOBECs will likely require the demonstration of an APOBEC3-related effect on endogenous retroelements in an organism.

### Lingering questions

There are still large gaps in our knowledge of non-LTR retrotransposon biology. Here I will discuss open questions that I feel are of outstanding interest. Since we are humans, and the non-LTR L1 element is that only known autonomous transposon in the human genome, these questions are framed in the context of mammalian L1 biology. However, some these questions can be addressed in various model systems, as cell biology between species is remarkably conserved.

#### What is the natural frequency of human retrotransposition?

'Back of an envelope' calculations have been used to estimate that, among every 10 to 20 new humans born, there is on average one new L1 retrotransposition event [143,144]. These calculations were based on multiplying the fraction of known human mutations caused by retrotransposition by an estimated total mutation rate per generation. Possible sources for error in these calculations are the underestimation of retrotransposition events due to detection bias and the reliance on phenotypes to find mutations, which only samples a subset of genes and genomic regions. Human retrotransposition frequency has also been estimated at 1 new insertion per 2 to 33 individuals, based on the summed retrotransposition frequency of tagged active L1s in a tissue culture or transgenic mouse retrotransposition assay [33,36,145]. Although consistent with previous estimates, these assays carry the caveat that they are performed in non-natural environments for a human retrotransposons (somatic cells and live mice). Current projects to catalog retrotransposon polymorphisms in humans will provide another way to estimate retrotransposition rates based on allele frequencies. However, the advent of next generation sequencing [146] and rapidly decreasing per base sequencing costs should make it possible in the near future to directly determine retrotransposition frequency by sequencing and comparing the genomes of successive family generations. This would not only give an average frequency of retrotransposition, but would also be able to highlight the potential wide variability of retrotransposition frequency between human individuals. This individual variability is due to variation of the number of 'hot' (highly active) L1s in a particular genome, as well as mutation of 'hot' L1s to give 'cool' alleles [147,148].

#### What pathways are at the LINE-host interfaces?

Previously, technical limitations have precluded laboratories from performing large systematic screens for factors that regulate LINEs. The studies leading to the recent exciting findings that small RNAs and APOBECs inhibit retroelements actually originated from non-retrotransposon laboratories. However, these serendipitous findings hint that careful examination will yield even more host pathways that interact with L1-like elements. In the past decade, the development of technology to knock down mammalian gene expression with short interfering RNAs [149] has made it possible to assess candidate gene contributions to retrotransposition without the time consuming step of manipulating mammalian chromosomes. This should allow the eventual screening of many, if not all, mammalian genes for effects on retrotransposition in standard tissue culture [33]. Simple yeast assays are also now available for LINE retrotransposition [150,151]. The fast and low cost methods of yeast genetics provide an attractive complementary system to probe the non-LTR/host interface.

Even with these technical advances, the limitations of current retrotransposition assays must be kept in mind when interpreting the results of a genetic screen. TPRT by definition involves breakage of DNA strands, and is therefore potentially genotoxic. The rate of L1 RNP formation, DNA damage, and stress (virtually every cell expressing active L1) far exceeds the rate of successful retrotransposition in a typical human cell culture assay (1 in 20 to 1 in 10,000) [79,152-154]. Therefore, one can easily imagine that any mutation that modifies the ability to tolerate DNA damage or stress will alter the apparent retrotransposition frequency, even if the true retrotransposition frequency remains unchanged. The retrotransposition assays are also not yet adapted for genetic screens in the natural L1 environment (germ cells). Although screens in embryonic stem cells [155] or sporulating yeast may come closer to approximating the natural context of LINE retrotransposition, the germ cell environment is quite unique and may contain non-somatic processes that stimulate or inhibit retrotransposition. Finally, unraveling the contribution of potential 'hits' to retrotransposition will be a daunting task, as the assay gives no indication as to what step in the poorly understand L1 life cycle is affected, and we still do not have a way to directly monitor which cells are undergoing active retrotransposition. This is perhaps not such a bad thing, as it will keep us occupied for years to come.

#### Localization of L1 proteins: what are we really looking at?

The vast excess of L1-containing cytoplasmic granules as compared to the actual number of successful retrotransposition events leads us to our next question. If TPRT occurs in the nucleus, why are RNPs overwhelmingly in the cytoplasm? Are we simply looking at non-functionally relevant, retrotransposon trash? An answer to this question is suggested by looking at the distribution of non-LTR retrotransposon RNPs in *Drosophila*. *Drosophila *has 20 families of non-LTR retrotransposons [156]. Rashkova *et al*. examined the location of five non-LTR element ORF1s, which presumably track with RNPs [48]. The *Het-A *and *TART *elements, which retrotranspose onto chromosome ends to form telomeres, are believed to perform an important cellular function for the host since *Drosophila *lacks telomerase enzyme. The RNPs for *Het-A *and *TART *were found to be efficiently transported into the nucleus. In contrast, RNPs for the euchromatic-targeted non-LTR retrotransposons *jockey*, *doc*, and *I *factor were predominantly sequestered in the cytoplasm. Thus it seems that host cells have a surveillance system that is able to distinguish 'friendly' from 'unfriendly' RNPs, and target only the latter for inhibition. It remains an open question whether these sequestered RNPs are still active and ready for retrotransposition, or marked for certain death. Is retrotransposition the result of a lucky RNP that escapes the stress granule, or is there an as of yet undetected 'true' retrotransposition pathway that bypasses these granules altogether? Is there a rare activating signal that can induce retrotransposition? Genetics, improvements in retrotransposition frequency, and the development of sensitive tools to visualize RNP transport and action in the nucleus will go a long way towards answering these questions.

#### Do we need retrotransposon activity?

A glance through genomes of higher eukaryotes will show something akin to 'genes floating on a sea of retrotransposons' [157]. How can these repeated sequences multiply to such high numbers if they do not provide some function for the host? A common mistake is to think of transposons as being under typical Mendelian constraints. This is not the case. Transposons can replicate and increase in number at a rate faster than single copy genes in the genome. In principle, an aggressively replicating transposon can rapidly sweep through a sexual population without conferring any fitness benefit [158]. The increase of transposon presence with each successive generation must simply outweigh any negative fitness effects.

Even if not conferring a net fitness benefit, transposons provide a source of endogenous mutation. Rather than simple point mutations, deletions and rearrangements, transposon mutations have the advantage of dispersing prebuilt functional units, such as transcription regulatory sequences, protein-binding DNA, and open reading frames encoding nucleic acid binding, cleavage, and synthesis activities. Thus, it is not surprising that after millions of years of evolution some of the many transposon bits and pieces littered among genomes have been put to use by the host cell. For example, genome rearrangements such as V(D)J recombination and programmed DNA deletion in ciliates are performed by proteins that share ancestry with transposases [159,160]. Retrotransposon sequences have also been incorporated into the coding sequence of many human proteins [161]. In addition, the silencing of retrotransposons can lead to epigenetic effects on adjacent genes [162]. The abundance of L1 elements on the X chromosome has even led to the theory that retrotransposons are involved in X inactivation spreading [163]. It is important, however, to note that host utilization of transposon remnants does not necessitate or even imply that active (retro)transposition is an important functional component of host cell biology. The only case of probable host requirement for retrotransposon activity that I am aware of is the aforementioned telomere maintenance of some insects, which is performed by non-LTR retrotransposons. Overall evidence suggests that transposons do not 'come in peace', and only after host adaptation are they subdued and in rare cases turned from the 'dark side'. Nevertheless, the presence of at least one case of a 'good' retrotransposon hints that there may be other interesting examples of symbiotic active retrotransposons awaiting discovery.

## Conclusions

The past 20 years has seen remarkable progress in our knowledge of non-LTR retrotransposon biology. Non-LTR retrotransposons are major components of eukaryotic genomes and our cells have evolved elaborate mechanisms to deal with these selfish elements. Since the L1 is the only active autonomous transposon in humans, and has directly or indirectly produced over one-third of our genome sequence, non-LTR retrotransposon biology is particularly important for understanding human genome evolution. The genotoxic effects of retrotransposons in mice and tissue culture also suggest that further investigation into the mechanisms of non-LTR retrotransposition will allow us to manipulate retrotransposon activity to confer potential health benefits. Finally, insight into host-retrotransposon interactions will give us a clearer picture on how cells recognize and silence DNA repeats; a question relevant to all of biology.

## Competing interests

The authors declare that they have no competing interests.
